# Research on Traffic Flow Prediction at Intersections Based on DT-TCN-Attention

**DOI:** 10.3390/s23156683

**Published:** 2023-07-26

**Authors:** Yulin Zhang, Ke Shang, Zhiwei Cui, Zihan Zhang, Feizhou Zhang

**Affiliations:** School of Earth and Space Sciences, Peking University, Beijing 100871, China; zhangyulin@stu.pku.edu.cn (Y.Z.); shangke@stu.pku.edu.cn (K.S.); zzh_cytus@pku.edu.cn (Z.Z.)

**Keywords:** traffic flow prediction, time convolution network, attention mechanism, digital twin, traffic saturation

## Abstract

Traditional nonintelligent signal control systems are typically used in road traffic signal systems, which cannot provide optimal guidance and have low traffic efficiency during rush hour. This study proposes a traffic signal phase dynamic timing optimization strategy based on a time convolution network and attention mechanism to improve traffic efficiency at intersections. The corresponding optimization was performed after predicting traffic conditions with different impacts using the digital twinning technique. This method uses a time-convolution network to extract the cross-time nonlinear characteristics of traffic data at road intersections. An attention mechanism was introduced to capture the relationship between the importance distribution and duration of the historical time series to predict the traffic flow at an intersection. The interpretability and prediction accuracy of the model was effectively improved. The model was tested using traffic flow data from a signalized intersection in Shangrao, Jiangxi Province, China. The experimental results indicate that the model generated by training has a strong learning ability for the temporal characteristics of traffic flow. The model has high prediction accuracy, good optimization results, and wide application prospects in different scenarios.

## 1. Introduction

An urban traffic signal control system is key to reducing traffic jams and accidents. With the rapid development of the scientific economy and an increase in population, the demand for motor vehicles is increasing in China. The traditional non-intelligent signal lamp is typically based on a fixed traffic signal control strategy, which makes it difficult to deal with the complex and changeable urban traffic state, leading to different degrees of traffic congestion and accidents, emissions of carbon dioxide, and huge economic losses [1].

In recent years, several studies have been conducted both domestically and internationally on urban traffic signal control issues, such as the split-cycle offset optimizing technique (SCOOT) system in the UK [2], real-time hierarchy optimized distribution effect system (RHODES) in the US [3], vehicle information and communication system (VICS) in Japan [4], and integrated traffic management system (ITMS) in China. To address the shortcomings of artificial experiences and sensor control, an increasing number of researchers have used artificial intelligence (AI) to improve urban transportation systems. Chin et al. [5] used Q-learning algorithms to control traffic lights, allowing each intersection to collaborate independently, while Casas et al. [6] used deep deterministic strategies for adaptive control of urban traffic signals. Kamal et al. [7] proposed a model predictive control (MPC) based on urban road traffic signal control; the MPC adapts to different traffic conditions and adjusts the free parameters of traffic signals online to generate appropriate traffic signals. Domestic scholars have also conducted studies on signal control. Xia [8] established an urban regional traffic signal control model to address the efficiency of traditional distributed adaptive traffic signal control coordination. He conducted traffic signal game interaction optimization control for local intersections and proposed a local information game interaction learning algorithm based on an intersection traffic signal control agent. Li et al. [9]. proposed a memory density strategy in which the NS-BML model in signal control only considers instantaneous density and ignores historical density. Based on the short-term memory density strategy combined with the long-term memory density strategy, the impact on a Manhattan-style network was analyzed to improve the operational efficiency of the road signal control system. Shi et al. [10] proposed a signal control strategy based on the operating characteristics of PFI traffic flow to address the congestion problem at urban traffic intersections and established corresponding optimization models, effectively improving the operational efficiency of intersections. Traditional adaptive signal control relies largely on the current traffic state and does not consider future traffic conditions, making it difficult to achieve an optimal global state. Researchers have applied methods such as neural networks [11], particle swarm optimization algorithms [12], and genetic algorithms [13] to adaptive traffic signal control (ATSC) to improve global optimization capabilities. However, these algorithms also have problems, such as the slow convergence speed of genetic algorithms and the low accuracy of particle swarm optimization algorithms. The research and development in the field of signal control is relatively recent in China, and its application must be improved through the application of science, technology, and industry standards. Signal control research has a high research value [14,15].

Based on the above analysis, this study proposes an optimized model for predicting traffic flow at the signal port based on a temporal convolutional network and attention with digital twins (DT-TCN Attention), fused with digital twin technology. The following are the contributions of this study.

(1) Using a TCN to extract temporal features from traffic flow data: A TCN introduces extended convolution and residual structures based on a convolutional neural network (CNN), which can effectively remove cross-temporal nonlinear relationships in traffic flow data. The residual system accelerates the feedback and convergence of the original multilayer neural network.

(2) Introducing an attention mechanism that allocates more weight to the temporal features of important nodes helps capture the global and local connections, which can effectively improve the interpretability and prediction accuracy of the model.

(3) In addition to considering the volume of traffic flow data, the study also determines meteorological factors, holidays, and seasonal information, which can help fully explore changes in traffic flow, improve the accuracy of prediction, and optimize the effectiveness of signal control.

(4) Digital twin software and Python tools were used to simulate the running status of vehicles in heavy traffic. By simulating intersections in practical cases and studying the coordinated timing control of signal lights at different periods, a real-time dynamic optimization strategy for each intersection phase was proposed to alleviate the congestion problem at peak traffic intersections.

The rest of the article is organized as follows: The related work is presented in Section 2 method of traffic Flow Prediction Strategy based on DT-TCN Attention. Following that, Section 3 introduces the data processing. Afterward, as a case study, the target system setup and model training are illustrated in Section 4. At the same time, based on the real-time dataset from traffic case studies, the evaluation results of the proposed model are discussed and presented in digital twin system. Discussion part is proposed in Section 5. Finally, conclusions and future research directions are outlined in Section 6.

## 2. Traffic Flow Prediction Strategy Based on DT-TCN Attention

### 2.1. TCN Attention Prediction Model Description

**Figure 1:**
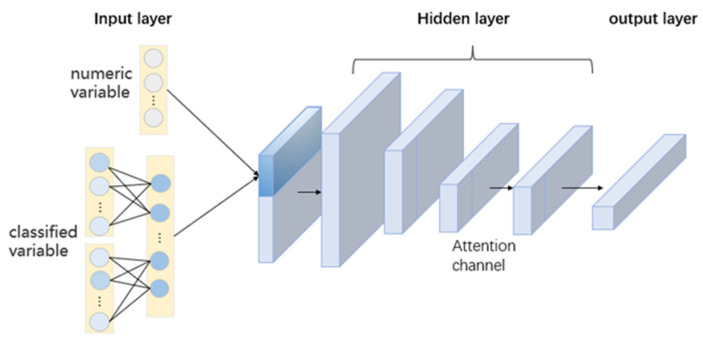
Traffic Flow Prediction Model Based on TCN Attention.

The traffic flow prediction model based on the TCN Attention, which is divided into three parts, is shown in Figure 1.

Input layer: The input layer contains the data pre-processing process, first embedding the special weather data and holiday data in the data set, and then importing the traffic flow data, and is set according to the corresponding variable characteristics of the data set for the integration of meaningless data truncation or supplementing it to ensure the integrity of the data set input.

Backbone network: Its function extracts nonlinear features from input sequences with multiple features.

Output layer: Outputs the multidimensional feature sequence output by the backbone network. The feature is extracted based on the backbone layer, and the training set data are used to abstract the feature and output the corresponding traffic forecast results in the future.

### 2.2. Data Preprocessing

In the daily long-timescale prediction task, the time-sampling unit preprocesses historical traffic flow, meteorological factors, holidays, and other data as input to the model, and the prediction result is the traffic flow operation in the next stage. The meteorological factors included the average temperature, wind speed, and maximum and minimum temperatures in the next step. The data preprocessing primarily includes data normalization, de-normalization, and categorical variable vectorization based on embedding.

#### 2.2.1. Data Normalization and Denormalization

Considering the significant differences between the original data and a large amount of data, data features with smaller values may need to be addressed. Therefore, the actual data in this study were normalized. This study adopts the normalization method of the linear function. The normalization formula is as follows:

$$x_{norm}=\frac{x-x_{\min}}{x_{\max}-x_{\min}}$$

where $x_{norm}$ is the normalized value obtained, *X* is the initial data, $x_{\min}$ represents the minimum value in the data, and $x_{\max}$ is the maximum value in the data. Normalize the original data to within [0, 1] to achieve proportional scaling of the original data.

After obtaining the predicted results from the short-term traffic flow prediction model of the TCN-Attention, performing denormalization processing was necessary. The denormalization formula is as follows:

$$x_{o}^{L}=x_{norm}^{L}(x_{\max}^{L}-x_{\min}^{L})+x_{\min}^{L}$$

where $x_{o}^{L}$ is the predicted value after inverse normalization, $x_{norm}^{L}$ predicts the output values for the model, and $x_{\max}^{L}$ and $x_{\min}^{L}$ are the maximum and minimum values in the original data, respectively.

#### 2.2.2. Vectorization of the Categorical Variable Based on Embedding

**Figure 2:**
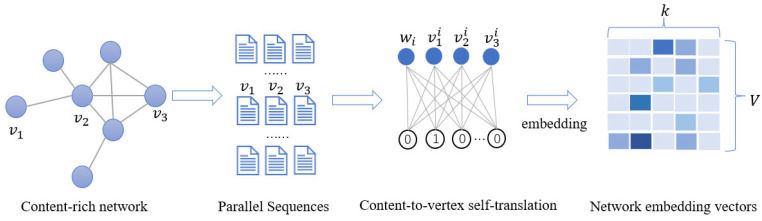
Embedded Layer Network Structure.

In this study, the one-hot code converted the categorical variable into a numerical value. For example, the one-hot code V for rainy days can be expressed as V = [1, 0, 0, 0]. To avoid using one-hot code that may cause deviations in the model prediction accuracy, the embedding layer network structure processes the categorical variable. As shown in Figure 2, high-dimensional sparse one-hot code V is converted into a low-dimensional dense embedding vector.

### 2.3. TCN Model Construction

The TCN model is an algorithm based on a convolutional structure (CNN) used to address time-series problems. It comprises a multilayer one-dimensional extended causal convolution and residual connected units. The input and output lengths are equal, and a residual connection mechanism is introduced to address the gradient vanishing problem.

**Figure 3:**
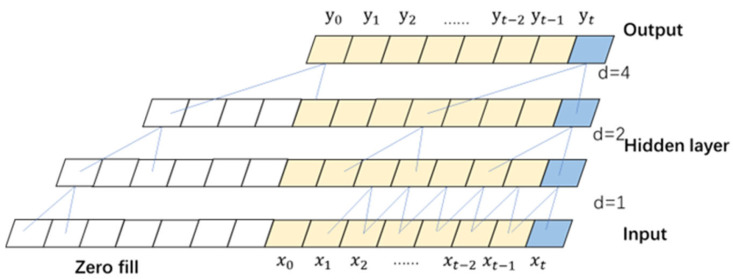
Extended Convolutional Network Structure.

The extended convolutional network structure selects the input of historical time $x_{0},x_{1},\ldots ,x_{t-1}$ as the input $x_{t}$ of the model t time, satisfies the input conditions of time-series prediction, and outputs $y_{0},y_{1},\ldots ,y_{t}$, which d are the expansion numbers, as shown in Figure 3.

The extended convolution sampled the input data in different steps with varying sampling frequencies at different levels. The input layer often samples all initial data to extract richer and more accurate feature information.

The residual unit was formed by adding the output and input data of different layers of the networks and the outputs after the activation function. The residual unit connection mechanism can improve the feedback and convergence of the network and avoid the problems of gradient disappearance and explosion that exist in traditional neural networks.

**Figure 4:**
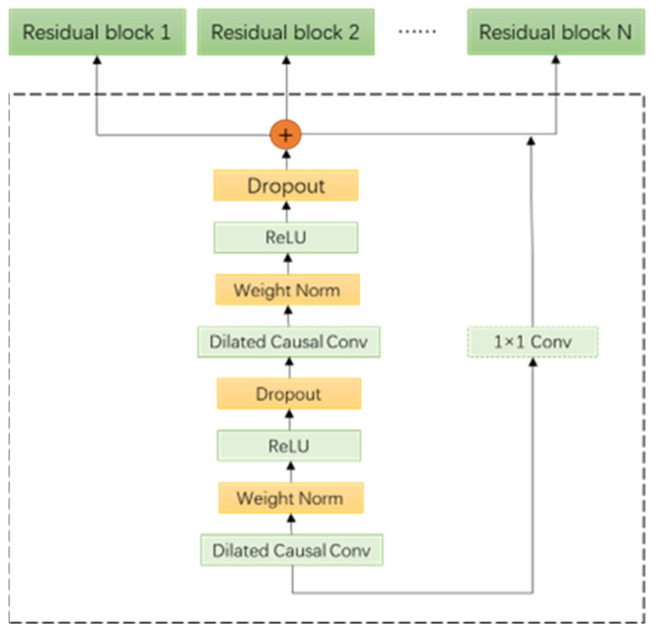
TCN residual unit structure.

Each residual element consists of two convolutional elements and a nonlinear mapping. First, the input data are subjected to one-dimensional extended causal convolution, and then the weight values are normalized to improve the calculation speed. The ReLU activation function is used to make the network nonlinear, and finally, dropout is performed on the output data, as shown in Figure 4.

### 2.4. Establishment of the Attention Model

The attention mechanism layer adopts a scaling and clicking method, and its output is

$$Attention(Q,K,V)=soft\max(\frac{QK^{T}}{\sqrt{d_{k}}})V$$

Here, K and V represent key-value pairs (key, value), Q represents the objective function, and $d_{k}$ represents Q of the dimension. If it requires Q=K=V to be satisfied, the calculated value of attention will be obtained.

**Figure 5:**
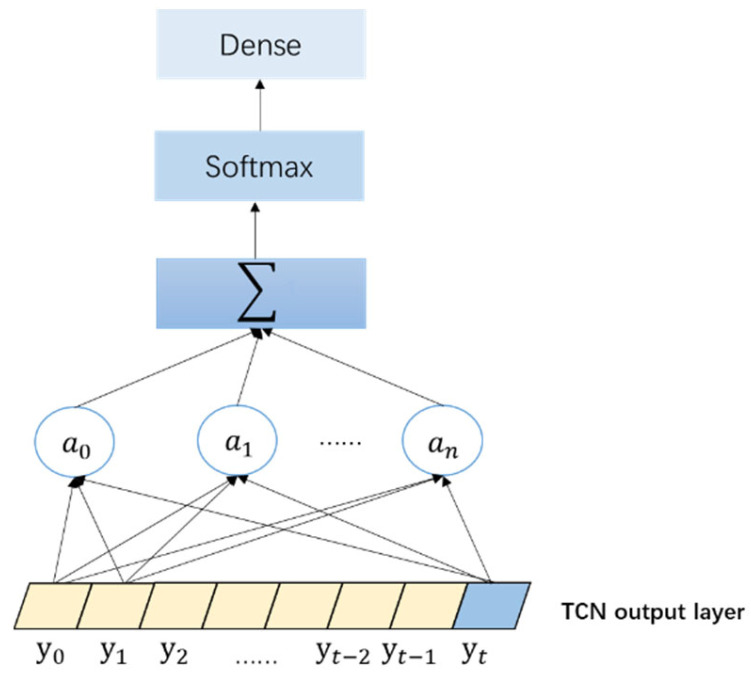
Attention Mechanism Network Structure.

The structure of the attention mechanism network is shown in Figure 5. The output value of the TCN output layer to the input of the attention mechanism layer was $a_{0},a_{1},\ldots ,a_{n}$. Different inputs had different features, and the features were significantly correlated with timing. The prediction results of each vector have different weights, and the attention mechanism performs a time-step action on values with higher weights in the prediction data. The weighted sum is used to obtain the attention value, and a dense output is obtained.

### 2.5. Digital Twin Signal Control System

A digital twin [16] refers to a digital representation of real-world entities. It integrates multi-scale and multi-dimensional simulations based on sensor data updates, historical data accumulation, and physical entity models. This reflects the life cycle of a physical entity. The digital twin [17] provides a new approach for analyzing and solving traffic signal timing problems by realistically restoring traffic operation scenarios in the digital space.

**Figure 6:**
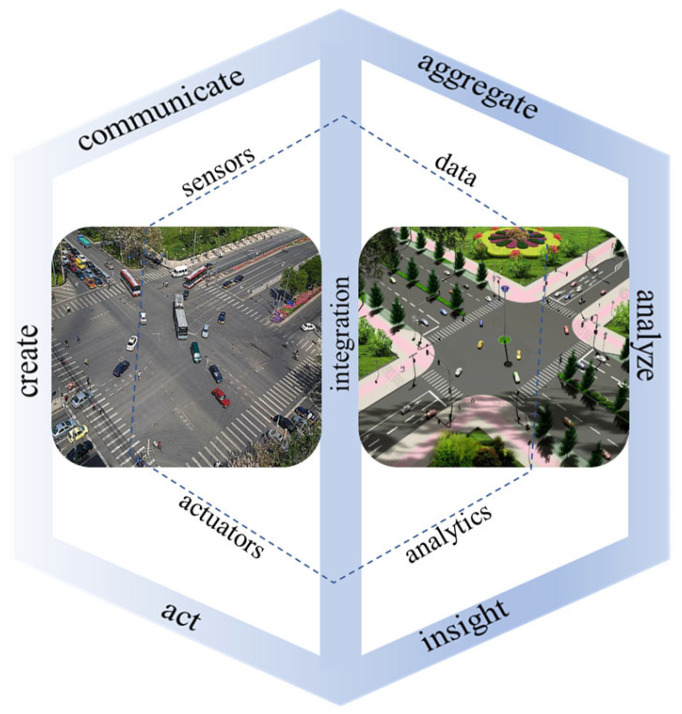
Structure diagram of the data interaction model for the digital twin signal control system.

The data access layer in the digital twin software can be used to build static models of road intersections and obtain dynamic vehicle data using sensor devices. The traffic signal control system has a fixed timing control for traffic lights that cannot be flexibly adjusted based on the actual traffic congestion situation without importing optimization algorithms. Real-time traffic data were imported into the twin simulation model, connected to digital analysis through the built-in COM interface in the digital twin software, optimized the algorithm timing of the traffic signal control model under twin construction and provided feedback on the optimized timing scheme in the real scene. The data interaction model structure of the digital twin signal control system is illustrated in Figure 6.

## 3. Data

The dataset in this study includes the effective traffic flow, meteorological factors, correlation dates, and nonhuman intervention factors from October 2022 to March 2023, as presented in Table 1. The pre-processing of raw data includes normalization, de-normalization, and embedding categorical variable vectorization. Among them, for effective traffic flow processing, the number of vehicles passing through and the busy index are calculated in units of 24 h per day. The processing of meteorological factors includes precipitation, wind intensity, and the daily temperature difference index. Non-human intervention factors primarily include traffic control and major vehicle accidents.

### 3.1. Traffic Flow Time Series

The dataset was sourced from the intersection of Fenghuang Middle Avenue and Shangrao Avenue in Shangrao City, Jiangxi Province, China. The data statistical period was from 14 October 2022, to 4 March 2023, and the statistical period was a day.

**Figure 7:**
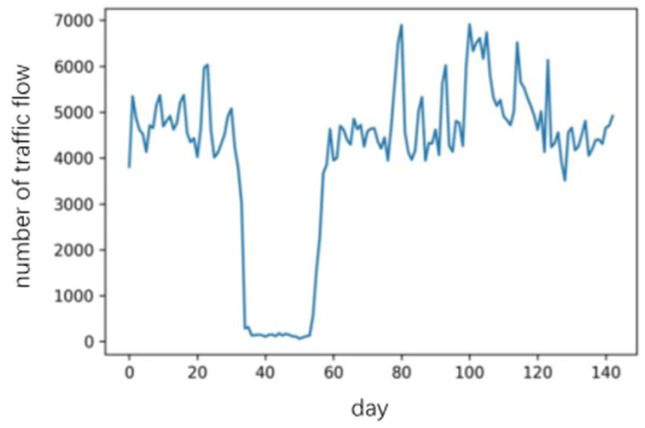
Dynamic Change Curve of the Dataset.

The dynamic change curve of the dataset is shown in Figure 7, where abnormal changes exist in the vehicle flow data during certain periods. The period from 20 November to 5 December was affected by pandemic policies, and the period from 21 January to 27 January was during the Spring Festival. The area is a population outflow area, and there are significant fluctuations in the trend of people returning home during the Spring Festival.

The daily traffic volume of this data set, which belongs to a traffic intersection with a large traffic volume, showed values above 5000 pcu over a long time. We note that the traffic flow fluctuates periodically, with about one large fluctuation every seven days, reaching a cyclical peak and a value of 6500–6800 pcu. Under the influence of the pandemic policy, the traffic flow had an obvious trough period. The figure was between 30 and 50 pcu for 14 days; traffic returned to normal after normal traffic policy resumed, and there were sharp spikes due to the holidays.

### 3.2. Impact Factors

A correlation exists between traffic flow data and morning and evening peak hours, holidays, and non-human-controllable factors. Therefore, based on embedding, this study transformed the relevant influencing factors of dynamic changes in traffic flow from text to low-dimensional vectors, defined as the time index, weather index, and non-human controllable index, as presented in Table 2.

**Figure 8:**
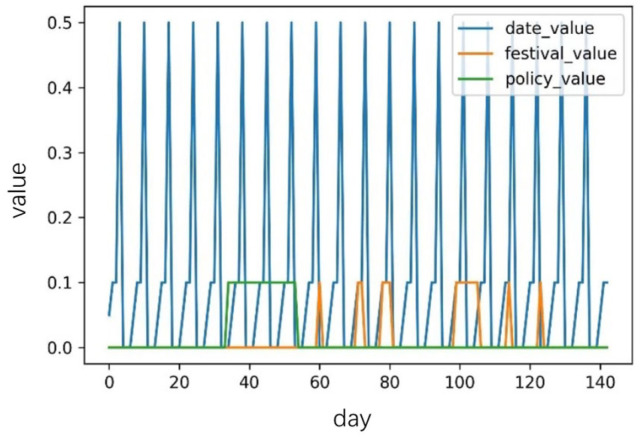
Parameter Weights of the Dataset.

The time index types were divided into three categories: “workdays not adjacent to holidays”, “workdays adjacent to holidays”, and “holidays/weekends,” with weights of 0, 0.05, and 0.1, respectively. A non-human controllable index, such as traffic control and congestion alleviation, primarily manifests as a temporary sharp drop in traffic flow. The weight during this period was set to 0.1, and the weight for the non-interference period was set to 0. A specific weight-parameter diagram is shown in Figure 8.

## 4. Experimental Verification

This study considers the intersection of Fenghuang Middle Avenue and Shangrao Avenue in Shangrao City, Jiangxi Province, from 1 December 2022, to 31 December 2022, and combines traffic flow survey data to train the TCN-attention model. The digital twin software was used for traffic simulation rehearsal and was compared with the TCN benchmark model to verify its effectiveness. The computer CPU used for all model training in the experiment is a 14-core Intel (R) Xeon (R) Gold 6330 CPU@2.00 GHz. The GPU is an RTX 3090 with 24 GB of graphics memory, and the operating environment is Python 3.8 (ubuntu18.04) + PyTorch 1.8.1 + Cuda11.1.

### 4.1. Evaluation Indicators

The simulation experiment uses the mean absolute percentage error (MAPE) to refer to the average absolute percentage error, a relative measure that determines the MAD scale as a percentage unit, rather than a variable unit. The average fundamental percentage error is a measure of the relative error, which uses actual values to avoid canceling the positive and negative errors. The formula used is as follows:

$$y_{MAPE}=\frac{1}{n}\sum_{t=1}^{n}\left|\frac{x_{real}-x_{pred}}{x_{real}}\right|$$

where $x_{real}$ represents the actual traffic flow, $x_{pred}$ is to predict traffic flow, and n is the number of predictions. The smaller $y_{MAPE}$ of the value, the closer the expected value is to the true value, which means the higher the accuracy of the prediction.

### 4.2. Adjustable Parameter Settings

The parameters of the TCN-attention traffic-flow prediction model include the embedding dimension, convolution kernel size, expansion coefficient, and number of residual blocks. The ReLU was selected as the activation function in the experiment, and the specific parameter settings are listed in Table 3. 

### 4.3. Network Training

**Figure 9:**
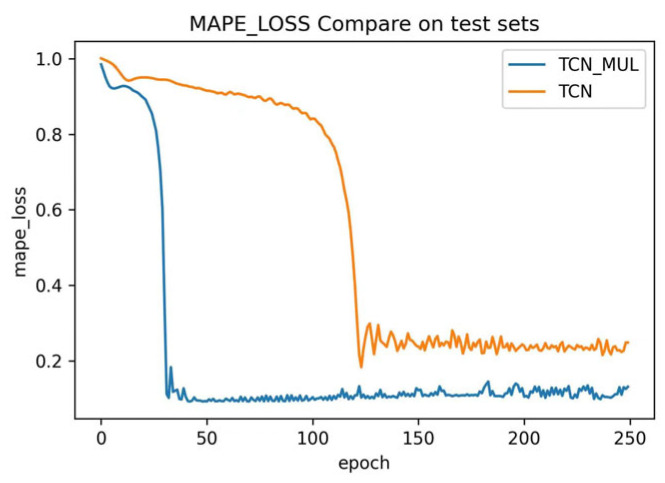
MAPE Error Curve of the Test Set.

**Figure 10:**
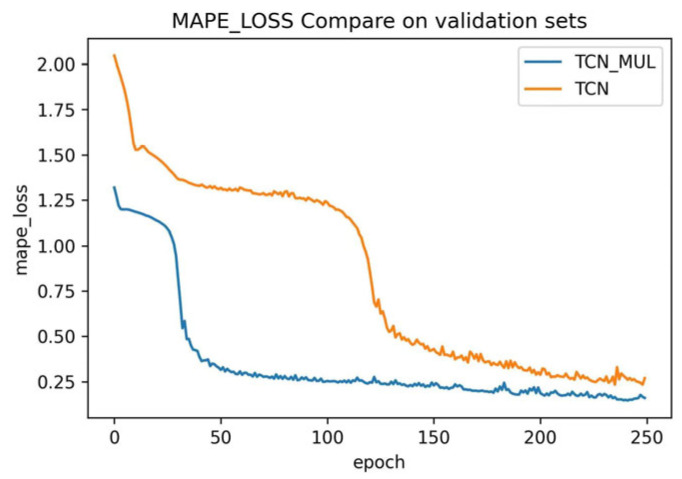
MAPE Error Curve of Verification Set.

The training used MAPE to analyze the prediction results and obtained the following results: The MAPE curves for the test and validation sets are shown in Figure 9 and Figure 10, respectively. After 250 epochs, MAPE of TCN and MAPE of TCN-Attention were 0.239 and 0.236 respectively, and the network converged.

It may be seen from Figure 9 and Figure 10 that in the TCN and TCN-Attention test sets, TCN-Attention converged much faster than TCN, and the MAPE values were consistently smaller. In the TCN and TCN-attention validation sets, TCN-Attention converged much faster than TCN as well. These results show that TCN-Attention is more effective than TCN’s traditional traffic flow prediction method, demonstrating the validity of the experiment.

**Figure 11:**
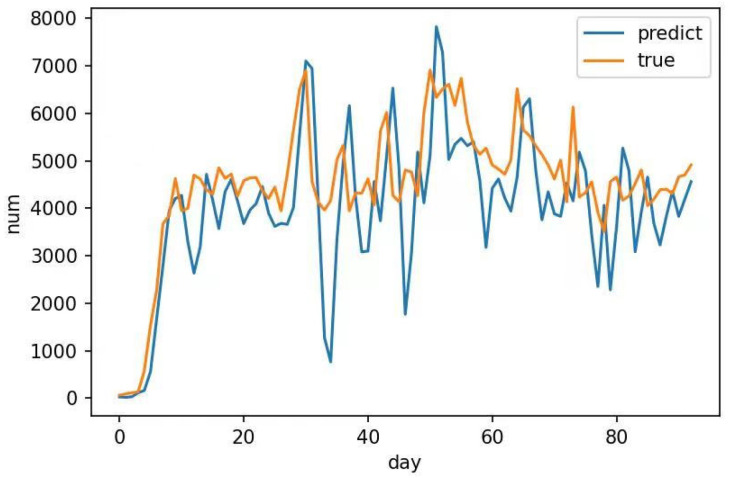
TCN Attention Prediction Results.

The final prediction results are shown in Figure 11:

(1) The overall trend of the network prediction results was consistent with the true values and could be adjusted for the initial stage and subsequent fluctuations. The prediction had relatively relevant fluctuations concerning the actual situation, and the prediction accuracy was good.
(2) The two predicted values in the middle were low and differed significantly from the true value. Owing to the inclusion of occasional variables related to the lockdown caused by the pandemic in the training set, they were included in network training. However, owing to the end of the pandemic and the resumption of normal traffic flow, a significant deviation occurred between the predicted and true values. Evidently, the subsequently predicted fluctuation amplitude gradually matches the true value.

### 4.4. DT-TCN Attention Run Test

Building the corresponding traffic network on the digital twin platform at the intersection of Fenghuang Middle Avenue and Shangrao Avenue in Shangrao City. The specific steps are

**Figure 12:**
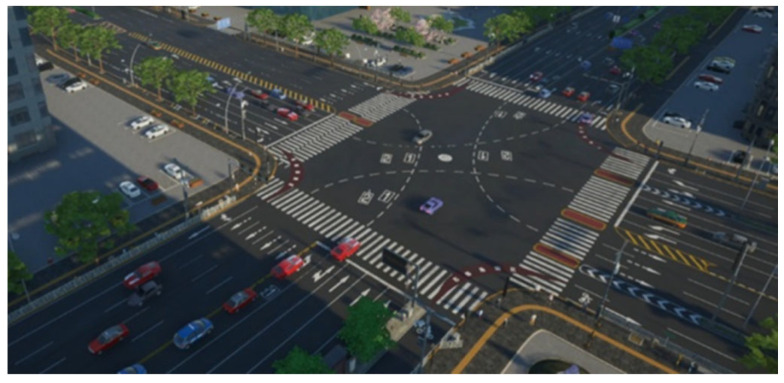
DT-TCN Attention Operation Effect Diagram.

1. Import the base map, perform precise positioning, and define the scale operation;
2. Draw 3D urban traffic sections and connectors;
3. Input traffic flow, place vehicle input modules at the corresponding entrances of the road network, and configure the type and quantity of vehicles;
4. Define the deceleration area of the road network and the path decision points;
5. Create a traffic signal controller to control the signal light groups at each intersection;
6. Place signal light groups and configure connectors at the parking line to calculate the duration adjustment of signal lights;
7. Construct an evaluation system, including establishing vehicle volume data collection points, travel time detectors, and queue length counters;
8. Import TCN Attention network training and run simulations internally.
9. The simulation results are shown in Figure 12, and the dynamic timing scheme is compared with the basic strategy presented in Table 4.

#### Signal Control Evaluation Indicators

In the study of intersection timing optimization, the evaluation indices include delay time, parking times, road capacity, saturation, fuel consumption, and exhaust emission. This paper selects the optimization objectives for the average delay, average parking, and queue length.

Vehicle delay consists of uniform delay and random delay. In this paper, a vehicle’s average delay time is calculated using the method of delay time proposed by Webster. The average vehicle delay $d_{i}$ for Phase i derived from the Webster delay calculation formula is as follows:

$$d_{i}=\frac{c{\left(1-\lambda_{i}\right)}^{2}}{2(1-y_{ij})}+\frac{x_{ij}^{2}}{2q_{ij}(1-y_{ij})}$$

In the formula, the first part represents the uniform delay, the second part the random delay; c represents the period, $\lambda_{i}$ is the green signal ratio, representing the ratio of the effective green light time of phase i to the signal period; $y_{ij}$ is the flow ratio of Phase i Inlet j, *X_ij_* is the saturation of Phase i Inlet j, and $q_{ij}$ is the actual traffic volume of Phase i Inlet j, pcu/h. According to the formula, the average delay of all vehicles at an intersection is expressed as:

$$\bar{d}=\sum_{i}d_{i}q_{i}/\sum_{i}q_{i}$$

The average number of stops at an intersection, averaged over a signal cycle by the total number of stops that a vehicle entering an intersection will produce under signal control, is expressed as:

$$\bar{h}=\sum_{i}h_{i}q_{i}/\sum_{i}q_{i},$$

where $h_{i}$ is the average number of stops for phase i vehicles.

Through a comparative analysis of the simulation evaluation results before and after, it can be concluded that the two signal timing optimization schemes have improved key indicators such as queue length and delay at intersections to varying degrees, improved traffic efficiency at intersections, and effectively alleviated traffic congestion. For the signal cycle optimization scheme under the Webster algorithm, the average queue length decreased by 6%, the average delay time decreased by 19.3%, and the average number of stops decreased by 0.6%. For the dynamic timing scheme based on the fusion DT-TCN Attention neural network, the average queue length decreased by 10.3%, the average delay time decreased by 27.1%, and the average number of stops decreased by 1.9%.

## 5. Discussion

As aforementioned, a neural network architecture integrating TCN Attention has the following advantages: parallelism, flexible receptive field size, stable gradients, low memory requirements during training, and variable-length input. This study used signal control optimization to effectively improve prediction accuracy. Further, digital twin technology is used for the simulation, effectively improving the driving efficiency. However, there are also obvious issues with this study.

1. The predicted results did not match the true values. In Figure 11, the predicted values represented by the blue line are consistent with the true values represented by the yellow line in the overall trend; however, a significant difference occurred in the position of the trough around 40 days. Owing to the consideration of the impact of pandemic policies (sporadic events) in the dataset, there was a period of less than 50 vehicles/day traffic data from the end of November to the beginning of December. Training was conducted during this period; therefore, there could have been significant fluctuation; however, the actual situation did not reflect this.

2. Potential parameter changes in the dataset. The characteristics of different datasets may have additional requirements regarding the historical quantity required for model prediction. Therefore, specific parameters must be adjusted for other datasets when using models for continuous predictions. This dataset includes the impact of policies and holidays. If the data volume increases for more than one year, other factors, such as weather, may affect the parameters. 

## 6. Conclusions

Owing to the increasing completeness of intelligent transportation systems, verifying traffic control strategies using digital twin simulation technologies has become important. To improve traffic efficiency at intersections, this study used digital twin software to predict traffic flow at road intersections and optimize the signal control simulation. The aim was to improve the effective green light time of each phase, overcome the limitations of target singularity, and provide a common simulation method for achieving traffic signal control.

This study integrated the TCN Attention neural network architecture to perform short-term traffic flow prediction on a time series in minutes and long-term prediction on a dataset in days. Considering multiple influencing factors such as related dates, holidays, and policies, the “Congestion Index” was proposed as an evaluation indicator. Experiments have shown that, based on traffic flow data at specific intersections, compared with traditional TCN, this method significantly improves accuracy and training efficiency, with MAPE = 0.239. By utilizing digital twin technology for real-time simulation and dynamically adjusting the signal control duration, it is evident that the delay time of the traffic flow is significantly decreased, yielding positive outcomes.

Additionally, the method proposed in this study requires improvements in certain aspects. In the case study analysis, this study only analyzed a single intersection and did not include any factors that affect pedestrian crossing time when considering factors that affect road traffic efficiency. With continuous improvements in computer technology, such as big data and cloud computing, the study of multiple factors, such as mixed traffic, can be added to future research. For each intersection phase, the model was established using the crossing times of pedestrians, electric vehicles, and cyclists as the objective function. Simultaneously, expanding the application scenario from simple single intersections to complex road network tests with multiple intersections and using the overall urban traffic scenario to reduce road congestion and traffic accidents is necessary.

## Figures and Tables

**Table 1:** Data Source Information.

Time Series Type	Time Interval	Start Date	End Date
traffic flow	1 day	14 October 2022	4 March 2023
Associated Date			
Meteorological factors			
Autonomous intervention			

**Table 2:** Types of influencing factors and parameter weights.

Index	Parameter	Factor
Time Index	workdays not adjacent to holidays	0.1
	workdays adjacent to holidays	0.05
	holidays/weekends	0.1
Non-human controllable index	none	0
	interference periods	0.1

**Table 3:** TCN-Attention Model Parameter Settings.

Parameter Name	Parameter Value	Parameter Name	Parameter Value
Embedding dimensions	3	Number of channels for each residual block	[24, 8, 8]
Convolutional kernel size	2	Channel compression rate	0.8
Number of residual block layers	6	Random inactivation rate	0.2
Coefficient of expansion	2	Batch size	512

**Table 4:** Comparison of intersection optimization algorithm timing schemes.

Timing Scheme	Cycle/s	Average Delay Time/s	Average Number of Stops	Average Queuing Length/m
Basic case	162	58.176	0.836	44.59
Webster	102	46.953	0.831	42.02
Dynamic timing	121	42.388	0.820	40.01

## Data Availability

Due to privacy, the datasets of this article is unavailable for publication.
